# Myopericarditis and Pericardial Effusion as the Initial Presentation of Systemic Lupus Erythematosus

**DOI:** 10.1155/2017/6912020

**Published:** 2017-02-05

**Authors:** Prema Bezwada, Ahmed Quadri, Atif Shaikh, Ceasar Ayala-Rodriguez, Stuart Green

**Affiliations:** ^1^Department of Medicine, The Brooklyn Hospital Center, Academic Affiliate of The Icahn School of Medicine at Mount Sinai, Clinical Affiliate of The Mount Sinai Hospital, 121 Dekalb Avenue, Brooklyn, NY 11201, USA; ^2^Department of Cardiology, Mount Sinai Beth Israel Hospital, 10 Nathan D Perlman Pl, New York, NY 10003, USA; ^3^Department of Cardiology, Mount Sinai Hospital, 1 Gustave Levy Pl, New York, NY 10029, USA

## Abstract

Myopericarditis with a pericardial effusion as the initial presenting feature of SLE is uncommon. We report an unusual case of myopericarditis and pericardial effusion with subsequent heart failure, as the initial manifestation of SLE. The timely recognition and early steroid administration are imperative in SLE-related myopericarditis with cardiomyopathy to prevent the mortality associated with this condition.

## 1. Introduction

Pericarditis is the most common cardiac manifestation of SLE [1]. Pericardial effusion is found in around 40% of SLE patients [2]. However, myocardial involvement with pericarditis, pericardial effusion, and subsequent heart failure, as the initial presentation of SLE, is uncommon. We describe an unusual case of myopericarditis with the development of pericardial effusion, cardiomyopathy, and heart failure as an initial manifestation of SLE in a young 35-year-old African American woman. She improved clinically with the resolution of myopericarditis and cardiomyopathy after treatment with methylprednisolone.

## 2. Case Report

A 35-year-old African American woman with a past medical history of an episode of pericarditis presented to the Emergency Department complaining of chest pain and fatigue of a weeks' duration. She reported a sudden onset of pleuritic chest pain associated with cold-like symptoms but no complaints of any fever or chills. In addition, she had reported difficulty lying supine, which caused some dyspnea along with some discomfort under the left breast. She had gone to the Emergency Department of another hospital, two days ago, and was discharged with the diagnosis of a viral syndrome. Upon further investigation, she reported a possible diagnosis of pericarditis when she went to another hospital in 2010. She then went on to give a history of hair loss and arthralgia in her hands. There was no other past medical history and family history was significant for SLE in a half-sister. She had 3 children and a 1st trimester miscarriage. She denied alcohol and drug use.

**Figure 1:**
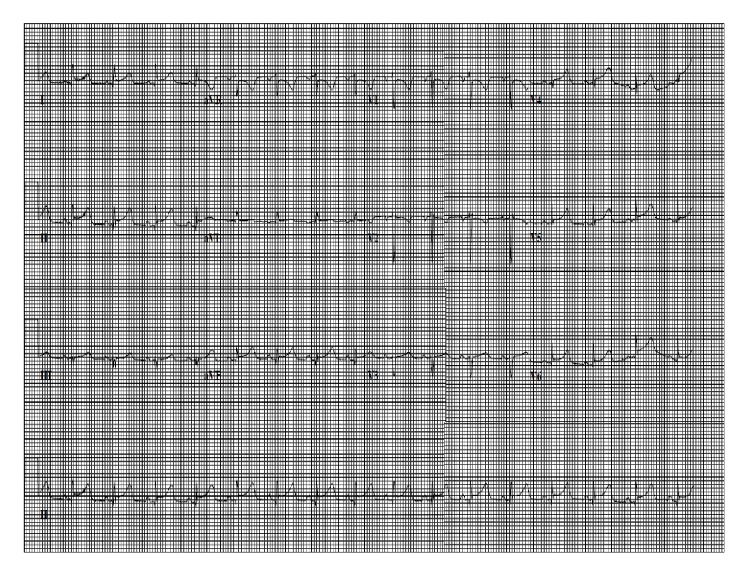
EKG on admission.

**Figure 2:**
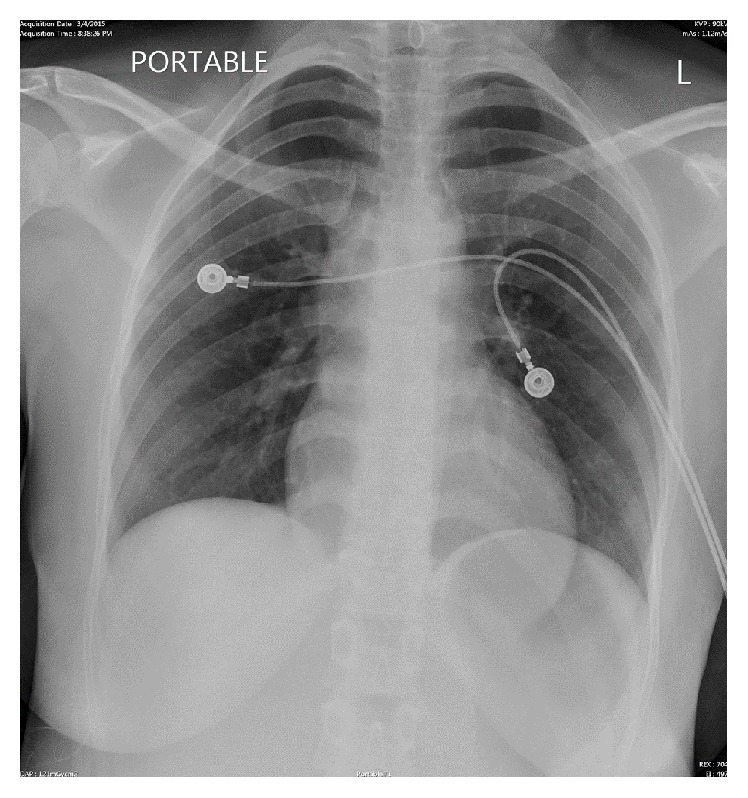
Chest X-ray on admission.

On admission, physical examination revealed a young woman with mild distress with tachycardia of 102. Other vital signs were within the normal limit. Only other significant findings on physical examination were a pericardial friction rub and nonscarring alopecia. Electrocardiogram (ECG) showed concave ST elevations in the inferior and lateral leads (Figure 1). Chest X-ray (CXR) showed no abnormalities (Figure 2). Labs including complete blood count and comprehensive metabolic panel were within normal limits. The first two sets of cardiac troponins were within normal limits. She was initially diagnosed as having pericarditis and was started on Indomethacin with Pantoprazole, but she could not tolerate NSAIDs. Hence, she was started on Colchicine with Prednisone. Additional labs, including antinuclear antibody (ANA), antidouble stranded DNA (Anti-ds DNA), and complement C3 and C4 were sent for further evaluation.

**Figure 3:**
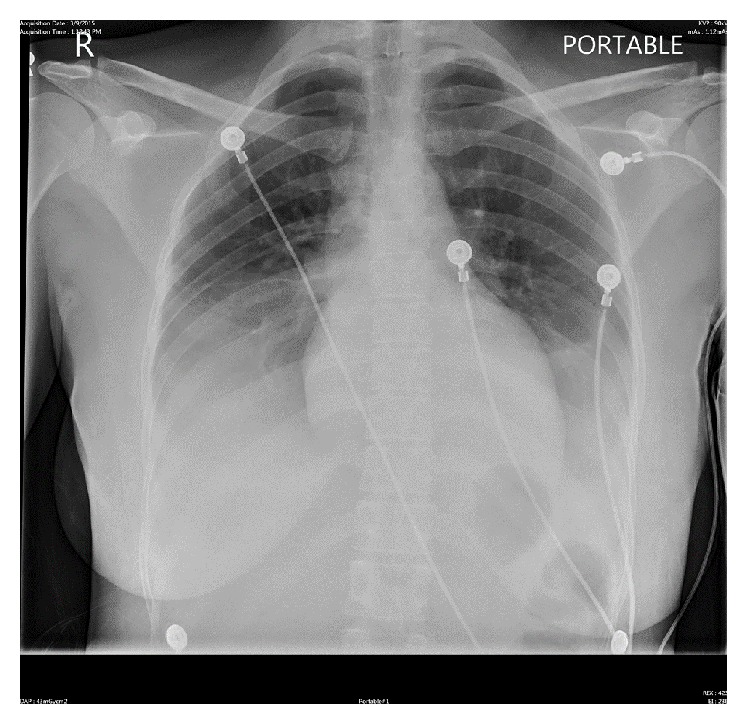
Repeated chest X-ray.

**Figure 4:**
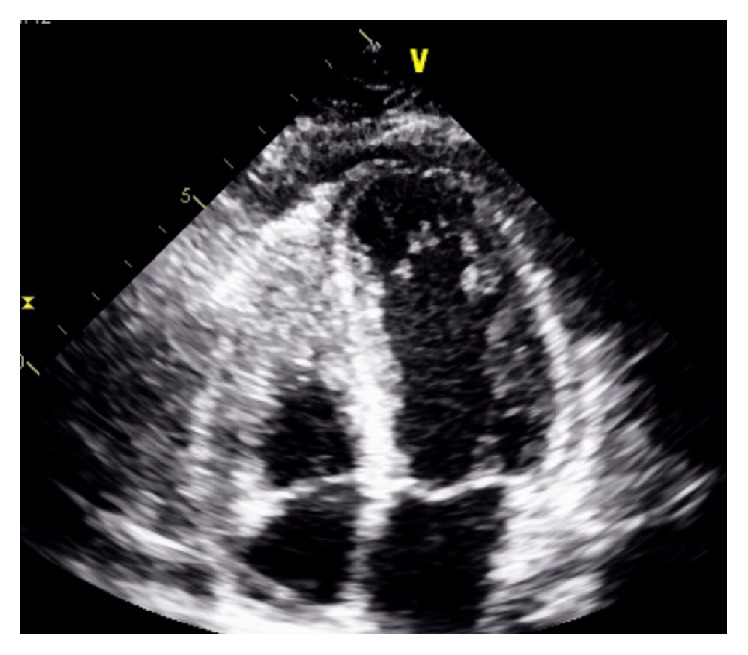
Transthoracic echocardiogram.

Later during the day, she complained of increasing shortness of breath with chest pain and found to be desaturating down to 87% on room air. She was noted to have crackles and trace pedal edema on physical examination. Arterial blood gas revealed hypoxemia. Her oxygen saturation improved after placing her on a nasal cannula with 2 liters of oxygen. A repeat chest X-ray showed an interval development of small bilateral pleural effusions (Figure 3). Repeat labs including Beta natriuretic peptide (BNP) and troponin were sent. A transthoracic echocardiogram (TTE) revealed moderately reduced left ventricular systolic function with an Ejection Fraction of 35–40% and a moderate pericardial effusion without any signs of tamponade (Figure 4).

The patient was transferred to the intensive care unit (ICU) for closer monitoring and started on lasix, metoprolol, and lisinopril. Troponin trend was 0.60 ng/ml > 0.49 ng/ml > 0.74 ng/ml, which was expected for myocarditis, and no heparin drip was indicated. She was very weak with marked dyspnea on exertion and could not get out of bed. Creatinine phosphokinase (CPK) was 435 U/L and BNP was elevated at 488 pg/ml. ANA returned positive at 1 : 160 with low C3 of 74 mg/dl and C4 of 25 mg/dl. SM/RNP, dsDNA, RPR, HIV, and HBsAg were negative. She was subsequently diagnosed with SLE, according to the new criteria, with pericarditis, myocarditis, and small pleural effusion but also ANA, low C3, and hair loss with alopecia. Prednisone was held and methylprednisolone was started. Colchicine was also held, as the patient developed diarrhea. Vitals remained stable with improvement in overall clinical status while the patient was in ICU but continued to feel weak. She was then transferred to a tertiary care center and had a cardiac magnetic resonance (CMR) imaging 3 days later, which showed normal biventricular size and systolic function, edema, and subepicardial enhancement in the lateral wall and confirmed the presence of myocarditis.

## 3. Discussion

Systemic lupus erythematosus (SLE) is classified as a systemic autoimmune disease, in which immune-complex depositions often result in an inflammatory response that involves many of the internal organs of the body, with an unpredictable flare-up and remission patterns.

Cardiac involvement has been reported to be one of the main complications contributing to the morbidity and mortality of patients suffering from systemic autoimmune diseases. While pericardial involvement is the most common echocardiographic lesion in systemic lupus erythematosus (SLE) and the most frequent cause of symptomatic cardiac disease, myocarditis remains fairly uncommon [3]. In clinical practice, myopericarditis is diagnosed by the presence of pericarditis with an elevation of cardiac markers or signs of myocardial inflammation [4].

Given our patient's history of recurrent pericarditis (first episode in 2010 and the current episode), the initial differential diagnoses included a viral etiology versus an autoimmune process, as she had initially reported an associated cough and cold-like symptoms and a family history, which included a half-sister, diagnosed with SLE. An autoimmune work revealed positive ANA and low C3 levels. Using lab results and further questioning, we were able to make a diagnosis of SLE based on the 2015 Systemic Lupus International Collaborating Clinics (SLICC) and American College of Rheumatology (ACR) revised criteria for the diagnosis of SLE [5]. Our patient had a total of 4 out of 16 points: nonscarring alopecia, pericarditis, low C3 levels, and a low titer positive ANA.

Echocardiographic findings are not specific to diagnose myocarditis in SLE, but global hypokinesis, in the absence of other known causes, is strongly suggestive [2]. Segmental areas of hypokinesis can also be suggestive of lupus myocarditis [6]. In our patient, a TTE initially showed a moderately impaired left ventricular systolic function with global hypokinesis and a moderate sized generalized pericardial effusion. A repeated TTE showed normal biventricular size and systolic function.

Another noninvasive test to diagnose lupus myocarditis is cardiac MRI (CMR) that usually shows gadolinium enhancement, especially in the subepicardial region with a patchy distribution. T2 values show myocardial relaxation abnormalities [7]. In our patient, CMR showed edema with subepicardial enhancement in the lateral wall and confirmed the presence of myocarditis. Cardiac MRI has been shown to be a very useful imaging modality in confirming the diagnosis of myocarditis. According to* the International Consensus Group on CMR Diagnosis of Myocarditis*, CMR may be used to identify patients with significant ongoing inflammation, especially for patients with recurrent or persisting symptoms and in patients with new onset heart failure [8]. Although an endomyocardial biopsy is widely considered to be the gold standard for diagnosing myocarditis, it is important to note its limitations and understand why a cardiac MRI is done instead. These limitations include exposing patients to higher risk of complications (perforation, tamponade) and the debate regarding the exact diagnostic criteria for analyzing myocardial tissue specimens [9].

Myocarditis in SLE has to be recognized and treated emergently with high-dose steroids. The addition of immunosuppressants as azathioprine or intravenous immunoglobulins (IVIG) may be helpful in treating myocarditis [10]. When our patient was started on high-dose IV methylprednisolone, she showed clinical improvement, with normalization of left ventricular systolic function on repeated TTE and CMR.

## 4. Conclusion

In conclusion, the combination of clinical presentation, laboratory tests, and CMR allowed us to make the diagnosis of myopericarditis secondary to SLE in our patient. Since myopericarditis with pericardial effusion was the initial presentation of SLE in our patient, she only improved after starting the steroids. The timely recognition and early steroid administration are imperative in SLE-related myopericarditis with cardiomyopathy to prevent the mortality associated with this condition. Therefore, a high level of clinical suspicion for SLE is necessary in patients presenting with pericarditis or myocarditis, in order to early diagnose and appropriately treat the underlying SLE.
